# Hypertrophic pyloric stenosis case series in twins and first cousins: genes, feeding patterns or both? (a case report)

**DOI:** 10.11604/pamj.2021.39.210.29180

**Published:** 2021-07-23

**Authors:** Konstantinos Skarentzos, Maria Aggelidou, Deftereos Savas, Konstantina Bekiaridou, Katerina Kambouri

**Affiliations:** 1 Democritus University of Thrace, Department of Medicine, General University Hospital of Alexandroupolis, Alexandroupolis, Greece,; 2 Democritus University of Thrace, Department of Paediatric Surgery, General University Hospital of Alexandroupolis, Alexandroupolis, Greece,; 3 Democritus University of Thrace, Department of Radiology, General University Hospital of Alexandroupolis, Alexandroupolis, Greece

**Keywords:** Pyloric stenosis, twins, first cousins, case report

## Abstract

We present two cases of dizygotic male twins and two cases of male first cousins with infantile hypertrophic pyloric stenosis (IHPS). All patients were treated with open pyloromyotomy. No complications were reported. The patients had the same risk factors for IHPS. First, all patients were first-born white males. Second, the twins were preterm (35 weeks). Third, the twins and the first cousins were exclusively bottle feeding. Thus, a combination of genetic and environmental factors may have contributed to the appearance of IHPS.

## Introduction

Infantile hypertrophic pyloric stenosis (IHPS) was first described by Hirschsprung in 1888 and is a common cause of gastrointestinal obstruction in newborns [1]. The pathophysiology of IHPS is characterized by smooth muscle cells that are not properly innervated. Smooth muscle cell relaxation is achieved by non-adrenergic, non-cholinergic nerves. Thus, it is believed that the absence of these nerves in the pyloric muscle causes contraction and hypertrophy of the muscle. Furthermore, the characteristic “firm” nature of the pyloric tumor may be explained by the excessive synthesis of collagen by circular muscle cells in IHPS. Additionally, the increased local synthesis of growth factors is attributed to muscle hypertrophy [2]. Since then, a great amount of research has been conducted regarding this disease, but the exact etiology remains unclear. In 1961, the hypothesis of the multifactorial threshold model of inheritance was suggested [3]. In recent years, environmental factors have been associated with IHPS. Children from a smoking mother have a higher risk of IHPS [4,5]. Pesticides have also been reported as a potential cause of IHPS [6]. Finally, bottle feeding is strongly associated with the disease [7]. Case reports of twins suffering from IHPS have been previously described in the literature, arguing whether genetics or environmental factors play a role in the etiology of IHPS [8,9]. The aim of this study was to present four cases of IHPS, two twins, and two first cousins that share some common environmental factors and investigate the etiology of IHPS.

## Patient and observation

**Patients´ information:** this study was designed according to the CARE checklist guidelines [10]. The first two cases were dizygotic male twins aged 25 days who presented to our clinic because of non-bilious vomiting after feeding. They were born after cesarean section by a healthy mother at 35 weeks of gestation. Their body weight was 3150gr g for A1 and 3320gr g for A2, and were exclusively bottle fed. Parental age was 30 years for mothers and 33 years for fathers. Physical examination revealed slide dehydration with decreased activity in both groups. She had no history of jaundice. The last two cases were two male first cousins aged 40 and 31 days, respectively, presented with a week interval to our clinic due to non-bilious vomiting after bottle feeding. Both were born with vaginal delivery from healthy mothers at 40 weeks of gestation. The first weight (B1) was 3200gr g, and its cousin (B2) weight was 3500gr g. B1´s parents aged: mother 23 years old and father 21 years old. B2´s parents aged: mother 16 years old and father 22 years old.

**Clinical findings:** biochemistry for A1 revealed serum urea (37 mg/dl), creatine (0.5 mg/dl) K+ (4.1meq/lt), Na+ (135meq/lt), cloride (84meq/lt), SGOT (48U/l), SGPT (22U/l). The biochemistry for A2 was as follows: serum urea (23 mg/dl), creatinine (0.4 mg/dl), K+ (3.3meq/lt), Na+ (134meq/lt), chloride (90meq/lt), SGOT (22U/l), and SGPT (30U/l). Ultrasonography of the abdomen showed for A1 and A2 pyloric canal lengths of 18 mm and 17.6 mm and wall thicknesses of 4.6 mm and 4.2 mm, respectively. Ultrasonography (US) findings were compatible with infantile hypertrophic pyloric stenosis (IHPS). No family history of IHPS was found in either parent. With the possible diagnosis of IHPS, abdominal US was performed for patients B1 and B2. The US findings for B1 were pyloric canal length (21.5 mm) and wall thickness (4.5 mm), and for B2 pyloric canal length (17.1 mm) and wall thickness (4.6 mm). There was no family history of IHPS.

**Therapeutic intervention:** each of these four patients was treated with open pyloromyotomy. No postoperative complications were reported in any of the cases. Specifically, after preoperative hydration and correction of electrolytes, the twins (first two cases) underwent open Fredet-Ramsted pyloromyotomy. The operation duration was 20 and 25 min, respectively, with a right upper abdominal incision almost 2.5 cm wide. Regarding the first cousins (last two cases) with all the biochemical markers and electrolytes under normal ranges, open pyloromyotomy was decided for both. The operation duration was 25 min for B1 and 21 min for B2 infants with a right upper abdominal incision approximately 2.5 cm long. The nasogastric tube was removed 8 hours after the operation.

**Follow-up and outcomes:** the postoperative course was uneventful for twins. Oral feeding began 8 h after the operation, and the babies normally discharged the 3^rd^ postoperative day. Regarding the first cousins, the postoperative course and feeding were uneventful. Both patients were discharged on the 3^rd^ day after the intervention. At the last follow-up, 6 months after the intervention, no complications were observed in any case.

## Discussion

Infantile hypertrophic pyloric stenosis is a common etiology of gastrointestinal obstruction in infants, with a prevalence of 20.09 per 10.000 live births. The incidence of IHPS is higher in non-Hispanic white males, first-born children, preterm births (<37 weeks), and infants from multiple gestations. Moreover, the rates of IHPS are higher for parents (father and mother) younger than 20 years of age at delivery. Furthermore, mothers with less than 12 years of education seem to have a higher prevalence of IHPS [11]. In 1961, Cartel *et al*. suggested a multifactorial threshold model of inheritance for IHPS [3]. The inheritance of congenital pyloric stenosis was thoroughly described in 1969. The proportion of co-twins affected by the disease was eight in 989. Regarding third-degree relatives, 25 of 2683 male cousins and 6 of 2580 female cousins were affected [12]. This makes our case series even rarer. The risk of IHPS is associated with a variety of genomic loci, such as BARX1 and EML4-MTA3 [13]. A recent study of metabolomics in IHPS concluded that even though IHPS is highly heritable, it does not have Mendelian transmission through families. Specifically, lower levels of certain metabolites were detected in IHPS patients, suggesting that feeding patterns in the first days of life may affect these metabolites [14]. Korgh *et al*. found that IHPS is associated with many pre- and perinatal factors and suggested that environmental factors may play a key role in the etiology of the disease [4]. In 2003, McAteer *et al*. reported an association between IHPS and bottle feeding [7]. The use of pesticides was highly correlated with IHPS [6]. So far, no infectious cause of IHPS has been identified. In 2010, McHeik *et al*. ruled out viral involvement in IHPS [15].

In this case series, we present four cases of pyloric stenosis. Specifically, two dizygotic male twins and two male first cousins. The patients had the same risk factors for IHPS. First, all patients were first-born white males. Second, the twins were preterm (35 weeks). Third, the twins and the first cousins were exclusively bottle feeding. Thus, a combination of genetic and environmental factors may have contributed to the appearance of IHPS. If we assume that both genes and environment lead to the disease, then a new question arises: to what extent does the environment affect the appearance of the disease and what can we do in order to prevent HIPS? In other words, does the effect of the environment play a crucial role in the onset of the disease in a way that we can prevent HIPS in the first place or do genetics dominate the course of the disease? More research with large groups of patients is needed to answer this question.

**Limitations:** this case series reports four cases of IHPS. The small number of patients limits our ability to extract generalized outcomes.

## Conclusion

Although IHPS is a common cause of gastrointestinal obstruction in newborns, its exact etiology remains unclear. In recent years, increasing numbers of environmental factors have been blamed for causing the disease. If the parents were informed about these environmental factors, the prevalence of IHPS would have been reduced.
